# Pol V produced RNA facilitates transposable element excision site repair in *Arabidopsis*

**DOI:** 10.17912/micropub.biology.000793

**Published:** 2023-05-16

**Authors:** Kaili Renken, Sarah M. Mendoza, Stephanie Diaz, R. Keith Slotkin, C. Nathan Hancock

**Affiliations:** 1 Biology and Geology, University of South Carolina Aiken, Aiken, South Carolina, United States; 2 Cardiovascular Disease Initiative, Bayer and Broad Institute of MIT and Harvard; 3 Donald Danforth Plant Science Center, St Louis, Missouri, United States; 4 Division of Biological Sciences, University of Missouri, Columbia, Missouri, United States

## Abstract

The plant-specific RNA Polymerase V (Pol V) plays a key role in gene silencing, but its role in repair of double stranded DNA breaks is unclear. Excision of the transposable element *mPing* creates double stranded breaks that are repaired by NHEJ. We measured *mPing* excision site repair in multiple DNA methylation mutants including *pol V* using an *mPing* : *GFP* reporter. Two independent mutant alleles of *pol V* showed less GFP expression, indicating that the Pol V protein plays a role in excision site repair. Sequence analysis of the *pol V* excision sites indicated an elevated rate of large deletions consistent with less efficient repair. These results clarify the role of Pol V, but not other RNA-directed DNA methylation proteins (Pol IV) or maintenance DNA methylation pathways ( MET1 ), in the repair of double-strand DNA breaks.

**Figure 1. mPing Excision Site Analysis:**
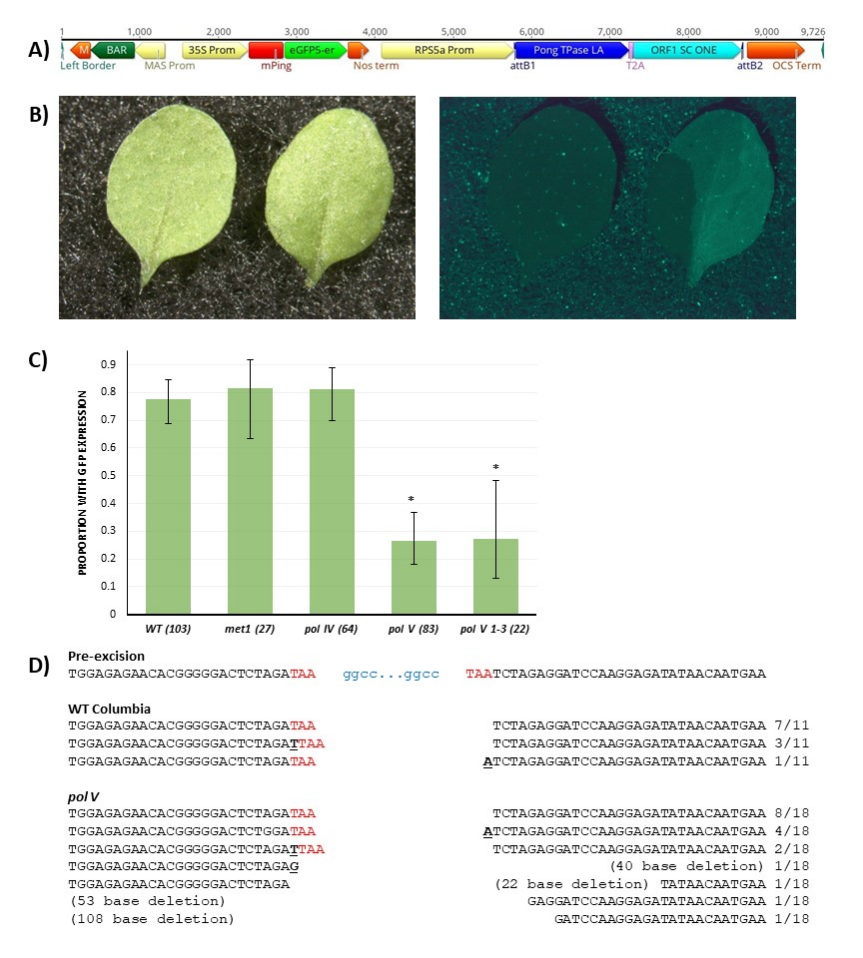
A. Diagram of *pEarleyGate100RMoA mPing* T-DNA that was used in *Arabidopsis* transformation. Promoters are shown in yellow; terminators are shown in orange. B. White light (left) and blue light (right) images of WT leaves with (right leaf) and without (left leaf) GFP expression induced by *mPing* excision. C. Graph of the proportion of *Arabidopsis* plants with GFP expression for each line tested. Axis labels are genotype (number of plants). Error bars indicate the standard deviation among each sample. * Indicates significantly different from WT, *met1* , and *pol IV* . D. Sequencing results for cloned excision site PCR products from WT and *pol V* plants. Pre-excision shows the location of the *mPing* element (blue) before transposition. The target site duplication bases are shown in red; insertions are underlined. Numbers on the right indicate the number of each sequence obtained/total number of sequences for that genotype.

## Description

Transposable elements are mobile sequences of DNA that are found in virtually all eukaryotic organisms and make up a sizeable percentage of the genome in some species, especially plants (Fedoroff 2012, Stitzer et al. 2021) . DNA transposable elements are excised and inserted by transposase proteins that cleave at the end of the elements, producing a double stranded DNA break (DSB) that must be repaired (Yuan and Wessler 2011, Hickman and Dyda 2016) . These DSBs are often repaired by the non-homologous end-joining (NHEJ) pathway, which often results in mutations at the excision site (Plasterk et al. 1999) . Previous studies indicate that small RNAs play a role in DSB repair in eukaryotes by potentially recruiting DNA repair enzymes or directly functioning as a repair template (Yang and Qi 2015, Bader et al. 2020, Franklin and Steele 2021) . Plants generate noncoding RNAs as part of the RNA-directed DNA Methylation (RdDM) pathway designed to silence repetitive sequences, especially transposable elements (Matzke and Mosher 2014) . Small interfering RNAs are produced by RNA Polymerase IV (Pol IV) and the plant specific RNA Polymerase V (Pol V) generates nascent scaffolding transcripts (Herr et al. 2005, Onodera et al. 2005, Lahmy et al. 2009) . *Arabidopsis* plants missing either of these RNA polymerases or the Dicer-like proteins that process the resulting RNAs were shown to exhibit reduced ability to repair a 35S:GU-US reporter gene cleaved between the repeated region with a restriction enzyme (Wei et al. 2012) . The repair of this reporter by homologous recombination was associated with the production of diRNAs (double-stranded break induced small RNAs) with homology to the sequences flanking the DSB (Wei et al. 2012) . A similar study showed that cleavage of the GU-US reporter by CRISPR also resulted in homologous 21nt small RNAs when it was highly expressed, but they were not detected from cleavage of a promoter-less GU-US construct or endogenous sequences (Miki et al. 2017) . In contradiction to the prior study, they didn’t detect a decrease in homologous recombination-mediated repair of the GU-US reporter in Dicer-like mutants (Miki et al. 2017) . Thus, there are still many unanswered questions about the role of RNA in DSB repair in plants.

*mPing* is an active miniature inverted repeat transposable element from rice (Jiang et al. 2003, Kikuchi et al. 2003, Nakazaki et al. 2003) that can be induced to transpose in *Arabidopsis* by expression of the ORF1 and Transposase (TPase) proteins from the related *Ping* or *Pong* elements (Yang et al. 2007) . Transposition frequency is measured using a *mPing* :GFP reporter ( Fig. 1A ) in which green fluorescent protein (GFP) is only expressed after *mPing* excision and the repair of the excision site (Yang et al. 2007) . Unlike the 35S:GU-US experiments described above, excision of *mPing* leaves behind compatible ends that are usually repaired by NHEJ, resulting in precise restoration to the state that would have been present before *mPing* insertion (Yang et al. 2007, Gilbert et al. 2015) . The purpose of these experiments was to test if the Pol IV ( AT1G63020 ) and Pol V ( AT2G40030 ) proteins are required for *mPing* excision site repair in *Arabidopsis* . As mutations of these genes result in alteration of DNA methylation, we also tested DNA repair in plants missing the main maintenance of DNA methylation gene, *MET1* ( AT5G49160 ).

*GFP* expression was measured in *met1, pol IV, pol V, pol V 1-3* , and Columbia (WT) *Arabidopsis* transformed with the *pEarleyGate100RMOA mPing* T-DNA ( Fig. 1A ). The *pol V* line used is a null allele while the *pol V 1-3* line is a single amino acid mutant that produces Pol V protein that is defective for RNA synthesis (Kanno et al. 2005, Lahmy et al. 2009) . We observed that ~80% of WT, *met1,* and *pol IV* had GFP expression, while the *pol V* and *pol V 1-3* lines had significantly lower percentage of plants with GFP expression [*p≤0.000137] ( Fig. 1B ). This indicates that in the absence of functional Pol V, either *mPing* excision frequency is reduced or the excision sites are not being repaired precisely by the *Pol V* mutants. *mPing* excision was detected in WT and *pol V* mutants using non-quantitative PCR with primers that flank the *mPing* excision site, suggesting that Pol V is not required for transposition. Sequence analysis of these excision site amplicons showed that 100% of the excision site repair in WT plants resulted in either precise repair or a 1 base insertion ( Fig. 1C ). In contrast, 22% of the excision sites in *pol V* plants had large deletions (>20 bp), with one deleting part of the promoter ( Fig. 1C ).

Together these results support a model in which RNAs produced by the Pol V protein play a role in NHEJ repair of the *mPing* excision sites. The finding that *pol V* and *pol V 1-3* mutants both exhibited the same decreased GFP expression indicates that Pol V produced transcripts and not simply the presence of the protein is required. In addition, excision site sequencing evidence suggest that abnormal repair is the cause of decreased GFP expression. The large deletions left behind in some *pol V* plants is consistent with stalled or slow NHEJ repair in which extra bases were excised from the ends. Our sequence analysis is also likely an underrepresentation of the quality of the excision site repair in *pol V* plants because PCR amplicons were only produced when the repair retained both primer binding sites.

Because the *mPing* excision site used in this study is directly after the 35S promoter, it is likely to be highly expressed similar to the 35S:GU-US reporter (Wei et al. 2012, Miki et al. 2017) . The 35S promoter has been shown to be a target for silencing mechanisms, including the Pol IV and Pol V mediated RdDM pathways (Mlotshwa et al. 2010, Li et al. 2012) . The observation that excision site repair was not significantly altered in other RdDM related mutants also provides important information. The fact that we did not see a difference in GFP expression in the *pol IV* knock-out tells us that the process of RdDM is not directly acting on NHEJ. Similarly, we can conclude that CG maintenance methylation is not responsible for DNA repair because the *met1* mutant had no change in GFP expression. Additional experiments will be needed to determine if the Dicer-like proteins play a role in the repair of the *mPing* excision site. It would also be interesting to test the repair of transposon excision sites that are not highly expressed or potentially targeted by RdDM pathways.

## Methods

*Arabidopsis Lines*

The KS019 ( *met1 allele “ddm2-1”* ) line was previously backcrossed multiple times into wild-type Columbia (Kankel et al. 2003) . Salk_083051 ( *pol IV* ), Salk_017795 ( *pol V* ), and CS69544 ( *pol V 1-3* ) were obtained from the Arabidopsis Biological Resource Center and genotyped by PCR and restriction digest analysis to confirm homozygosity. The genotype of CS69544 ( *pol V 1-3* ) was confirmed by sequencing the PCR amplicon from the following primers: AT2G40030 1317 For – 5’ CCCTCTGATGTGTAGCCCCCTCAG and AT2G40030 1627 Rev – 5’ GGAAAACTGTCCAAGCTGGGCCAG. Young plants were grown at 23°C in 10 hours of light per day, mature plants were grown at 25°C with constant light.

*Construct development and plant transformation*

The 35S promoter in pEarleyGate 100 (Earley et al. 2006) between the *Xho* I and *Stu* I sites was replaced with the RPS5a promoter and then the Pong TPase L418A, L420A/T2A/ORF1SC1 ONE construct was gateway cloned into the plasmid. The *mPing* :e *GFP* reporter from pBIN (Yang et al. 2007) was cloned into the *Bgl* II site by ligation. The sequence verified construct was transferred to GV3101 *Agrobacterium* and *Arabidopsis* transformation was performed by floral dip (Clough and Bent 1998) . Transgenic seedlings were selected by spraying daily with a 1:500 dilution of Liberty herbicide before being transplanted to new flats.

*GFP screening*

Fluorescent microscopy was performed on 3–4-week-old seedlings using a Leicia M165 FC dissecting microscope equipped with a pE-300 ^lite^ LED light source and an ET GFP - M205FA/M165FC filter. Plants were visualized by two individuals and classified as having GFP expression in a binary (+/-) process. Fluorescence that results from transposition is usually localized to sectors or large spots on the leaves (Yang et al. 2007) , allowing us to differentiate from other fluorescent sources.

*DNA Analysis*

*Arabidopsis* DNA was extracted using the CTAB method. Briefly, a young leaf was ground in 600 μl of CTAB buffer (1% CTAB, 0.1 M Tris pH 8, 0.02 M EDTA, 5 mM ascorbic acid, 0.02% PVP-40, 4 mM DIECA) and incubated at 65°C for 30 minutes before adding 400 μl of chloroform. After centrifugation, the supernatant was transferred to a new tube and precipitated with 300 μl isopropanol, washed with 70% EtOH, dried, then resuspended in 50 μl of TE. PCR was performed using the pBIN Flank For – 5’ AGACGTTCCAACCACGTCTTCAAAGCAA and pBIN Flank Rev – 5’ CCTCTCCACTGACAGAAAATTTGTGCCCA primers with the following conditions: 30 cycles of 95°C for 30 sec, 58°C for 30 sec, and 72°C for 90 sec. The bands containing the excision sites were gel purified and cloned into pGEM® T Easy (Promega) before Sanger sequencing. The sequence results were aligned to the expected template for precise *mPing* excision site repair using Geneious Prime 2022.2.2 software (https://www.geneious.com).

## Reagents

**Table:** 

**STRAIN**	**GENOTYPE**	**AVAILABLE FROM**
CS70000	Wild type Columbia	ABRC
KS019	*met1* ( *ddm2-1* allele)	Keith Slotkin Laboratory
Salk-083051	*pol IV*	ABRC
Salk-017795	*pol V*	ABRC
CS69544	*pol V 1-3*	ABRC

**Table:** 

**PLASMID**	**GENOTYPE**	**DESCRIPTION**
pEarleyGate 100RMOA *mPing*	Rps5a: *Pong* TPase L418A, L420A/T2A/ORF1SC1 ONE, 35S: *mPing* : *eGFP*	Addgene #196137
